# Utilizing a Novel AI Tool to Detect the Posterior Superior Alveolar Artery’s Location’s Impact on Maxillary Sinus Mucosal Thickening in the Presence of Periapical Lesions

**DOI:** 10.3390/medicina60010140

**Published:** 2024-01-12

**Authors:** Wael Aboelmaaty, Abdulmohsen Alfadley, Mohammed Awawdeh, Ahmed Saaduddin Sapri, Lama Awawdeh, Eman Shawky Mira

**Affiliations:** 1 Maxillofacial Surgery and Diagnostic Sciences Department, College of Dentistry, King Saud bin Abdulaziz University for Health Sciences (KSAU-HS), Riyadh 11426, Saudi Arabia; 2 Oral Radiology and Diagnostic Sciences Department, Faculty of Dentistry, Mansoura University, Mansoura City 35516, Egypt; emanshawky@mans.edu.eg; 3 Dental Services, King Abdulaziz Medical City, Ministry of the National Guard—Health Affairs, Riyadh 11426, Saudi Arabia; fadleya@ksau-hs.edu.sa (A.A.); awawdehm@ksau-hs.edu.sa (M.A.); 4 King Abdullah International Medical Research Center, Ministry of National Guard—Health Affairs, Riyadh 11481, Saudi Arabia; 5 Department of Restorative and Prosthetic Dental Sciences, College of Dentistry, King Saud bin Abdulaziz University for Health Sciences, Riyadh 11426, Saudi Arabia; 6 Preventive Dental Science Department, College of Dentistry, King Saud bin Abdulaziz University for Health Sciences (KSAU-HS), Riyadh 11426, Saudi Arabia; 7 College of Medicine & Dentistry, Ulster University, Birmingham B4 6BN, UK; 8 Division of Oral and Maxillofacial Surgery, Batterjee Medical College, Jeddah 21442, Saudi Arabia; ahmedsaaduddin@mans.edu.eg; 9 Oral and Maxillofacial Surgery Department, Faculty of Dentistry, Mansoura University, Mansoura City 35516, Egypt; 10 Department of Conservative Dentistry, Faculty of Dentistry, Jordan University of Science and Technology, Irbid 22110, Jordan; lawawdeh@hotmail.com

**Keywords:** PSAA location, mucosal thickening, AI tool, eVol DXS

## Abstract

Periapical lesions have been implicated in sinus-related complications, but the precise influence of anatomical variations in the posterior superior alveolar artery (PSAA) on mucosal thickening remains an uncharted aspect. The new AI tool employed in this research utilizes advanced image processing algorithms to enhance image visualization. *Background and Objectives*: This study examines the accuracy of a new cone beam computed tomography (CBCT) software (eVol DXS, version 1.0.1.0) employing AI to detect the PSAA’s location and the effect of that on maxillary sinus thickening in the presence of periapical lesions. *Materials and Methods:* This retrospective study included 120 CBCT cases with posterior maxillary periapical lesions and 120 without odontogenic infections. Teeth with proximity (<2 mm) to the sinus were excluded in both groups to eliminate the sinus floor’s perforation effect. Both the PSAA locations and maxillary sinus thickening were classified and compared. *Results*: The mucosal thickening differs significantly (*p* < 0.001) between the study group and the control group. The study showed that an increased sinus thickness occurred when the PSAA was beneath the sinus membrane in the study group (62.5% compared to 8.6%; *p* < 0.001 *). The AI tool helped to achieve a 100% identification rate in determining the PSAA locations. *Conclusions:* AI algorithms for PSAA localization, which affects mucosal thickness in response to periapical lesions, yield excellent results.

## 1. Introduction

The thickening of the mucosal lining of the maxillary sinus pertains to an atypical augmentation in the thickness of the mucous membrane that lines the sinus cavity. The thickening of the nasal passages can be attributed to a range of reasons, including sinusitis, allergies, nasal or oral infections, the growth of nasal polyps, exposure to irritants such as smoking or pollution, gastroesophageal reflux disease, and some immune system problems [1]. The correlation between periapical lesions and maxillary sinus mucosal thickening is a multifaceted interaction that encompasses different anatomical and pathological factors. Periapical lesions are frequently linked to endodontic infections and can potentially impact neighbouring structures such as the maxillary sinus. The condition has the potential to induce localized inflammation and eventual hypertrophy of the sinus mucosa [2]. This phenomenon may also be influenced by anatomical abnormalities, such as the proximity of the roots of the posterior maxillary teeth and the communication between periapical lesions and the sinus, particularly in cases with oroantral involvement [3].

The posterior superior alveolar artery (PSAA) is a terminal branch of the maxillary artery. It is responsible for supplying blood to many anatomical structures, including the maxillary sinus, the maxillary premolars and molars, as well as some regions of the buccal gingiva [4]. As this artery reaches the maxilla through the posterior superior alveolar foramen, it passes next to the maxillary nerve’s posterior superior alveolar branch, which supplies the upper molars [5]. The terminal connection between the PSAA and the branches of the infraorbital artery occurs as the vessel traverses the sinus. This process leads to the emergence of both an interosseous and an extraosseous branch [6].

Most variations in the PSAA feature the artery snaking its way through the sinuses and underlying bone. While the predominant occurrence of the PSAA is within the maxilla, there have been documented instances of its presence in the mucosa of the maxillary sinus. In exceedingly rare cases, it has been reported to be located superficially to the mucosa or externally to the lateral wall of the maxillary sinus [7,8].

In order to minimize the risk of arterial injury and consequent perioperative bleeding, it is imperative to accurately identify the precise location of these arteries prior to undergoing sinus floor augmentation surgery [9]. Perforation of the maxillary sinus membrane commonly occurs during the process of dental implant installation. Bleeding is a frequently encountered complication of sinus lift surgery, ranking second in prevalence only to Schneiderian membrane puncture. Most of the bleeding is a direct result of the PSAA being injured [10]. An insufficient understanding of PSAA anatomical variations, the osseous composition of the maxilla, and the Posterior Superior Alveolar Nerve (PSAN), along with their respective anatomical characteristics, can lead to severe difficulties during various surgical interventions, particularly in the context of dental implant placement [11].

The utilization of three-dimensional imaging technology to enhance our comprehension of the maxillary sinus structure can be quite beneficial. Several factors must be taken into account while selecting the suitable radiography method for each particular patient [12]. In the context of medical imaging, it has been observed that the utilization of cone beam computed tomography (CBCT) results in a notable reduction in the radiation dose experienced by the patient when compared to conventional computed tomography (CT) [13]. In recent years, there have been significant technological advances, resulting in the attainment of much higher levels of resolution in dental imaging [14].

It is important to note that the ability of CBCT to identify the artery was observed in the range of 60.58% to 87% among the tested groups. This is due to the fact that the detection of the PSAA presents greater challenges, particularly among patients who are completely toothless. Approximately 33% of cases provide challenges in effectively detecting or locating the PSAA [15]. 

The utilization of artificial intelligence (AI) in the enhancement of radiographic images has demonstrated considerable promise in enhancing the identification and visualization of anatomical components within the maxillofacial region. A wide range of AI techniques and algorithms can be employed to augment the caliber of radiography images, facilitating healthcare practitioners in their task of effectively interpreting and diagnosing medical disorders. These enhancements include noise reduction, contrast enhancement, artefact removal, automated segmentation, and custom clinical tasks [16]. AI plays a pivotal role in the field of radiographic image enhancement, as it utilizes sophisticated algorithms and filters. Convolutional neural networks (CNNs) and deep learning architectures are used in AI-driven image processing approaches to evaluate radiography data in a complex manner. These algorithms have the capability to accurately detect and reduce unwanted disturbances, resulting in enhanced ratios between the desired signal and the background noise. In addition, AI utilizes contrast enhancement algorithms to amplify tiny differences in tissue density, thus improving the clarity of anatomical features [17].

In addition, AI-powered filters, such as edge-preserving filters and adaptive histogram equalization, enhance the quality of the image details. Edge-preserving filters, such as bilateral and anisotropic diffusion filters, enhance the edges while maintaining smooth regions, leading to the improved delineation of structures. Adaptive histogram equalization modifies the image histogram in a localized manner, highlighting certain areas of interest and resolving the problems associated with uneven lighting. The combination of these techniques not only enables the recognition of anatomical features but also aids in the prompt diagnosis of problems. AI streamlines the image enhancement process, allowing radiologists to concentrate on interpretation and diagnosis. This enhances the workflow efficiency and boosts the diagnostic accuracy in medical imaging. The ongoing progress of technology enables the incorporation of AI into radiography, which has the potential to consistently enhance the quality of images and diagnostic abilities in the medical imaging domain [17]. Evaluation of the accuracy of these tools is important to determine their clinical value during dental practice. A review of the existing literature has revealed a scarcity of studies on the correlation between the anatomical position of the PSAA and the severity of maxillary sinus mucosal thickening. Apart from a single study by Yalcin and Akyol (2019) that explored the link between PSAA prevalence and susceptibility to maxillary sinus pathologies [18], there has been no previous research investigating this specific relationship. The current study aimed to assess the accuracy of a new CBCT software tool (eVol DXS software, version 1.0.1.0, CDT Company, São José dos Campos, Brazil) which uses AI advanced filters for image enhancement to detect the location of the PSAA. Also, it has evaluated the effect of the PSAA location on the degree and extent of maxillary sinus thickening in relation to odontogenic periapical lesions.

## 2. Materials and Methods

### 2.1. Study Sample

This is a retrospective cross-sectional study. It has utilized the records of a sample of patients referred from outpatient clinics at the Faculty of Dentistry, Mansoura University, Egypt, for CBCT scanning. The study sample included CBCT performed between January 2018 and January 2023. A total of 120 CBCT scans of patients with a written report of inflammatory periapical lesions associated with non-vital teeth or endodontically treated maxillary premolars and molars were selected in this study for further analysis. Any history of dentoalveolar surgery, trauma, fracture, metabolic disorders, chronic maxillary sinusitis, or sinus surgery prompted exclusion from this study. Furthermore, the selection of an additional 120 CBCT scans with no history or reported radiograph of any inflammatory disease relating to the maxillary premolars, molars, or maxillary sinus acted as a control group. Each one of the selected cases had to meet the criterion that the roots of the premolars or molars had a minimum of 2 mm distance from the floor of the sinus with no sinus floor involvement. This ensured that the roots’ tips were not perforating the sinus floor and affecting the mucosal thickness. The confidentiality of the patients was maintained by exporting all the CBCT volumes anonymously so that all personal data remained confidential. 

Assuming that 5% of the subjects in the reference population have the factor of interest, and after applying continuity correction, the study would require a sample size of 119 for each group (i.e., a total sample size of 238, assuming equal group sizes) to achieve a power of 80% for detecting a difference in proportions of 0.12 between the two groups (test–reference group) at a two-sided *p*-value of 0.05 with a 95% confidence level. Hence, the sample size was rounded to 120 in each group. Consecutive non-probability sampling was adopted until the required sample size was reached. The examination was conducted by two independent experienced radiologists. The raters were calibrated with excellent interrater reliability (Cohen’s kappa coefficient = 0.92). Any disagreement was discussed between the raters until a consensus was reached.

### 2.2. Radiographic Analysis

The CBCT images were taken using multiple fields of view according to the type and the extent of inflammatory lesion, with a voxel size of 0.25 mm, voltage of 120 kV, and 8 mAs. The CBCT cases were classified into two groups: the study group, which had 120 CBCT cases with inflammatory periapical lesions with non-vital teeth, and the control group, which had 120 CBCT cases free from any pathological conditions in the maxillary premolar and molar region. All the volumes were oriented carefully and then sliced at a 1 mm slice thickness in the orthogonal plane view. Coronal and sagittal cuts were used for the PSAA evaluation.

All 240 CBCT images were evaluated using the new specialized AI tool (eVol DXS software, CDT company, São José dos Campos, Brazil) to detect the presence and location of the PSAA. This software uses new advanced clinically customized 3D filters applied to a single-slice 3D image using AI technology for image enhancement. The location of the PSAA was detected in all groups and classified into IO = intraosseous, BM = beneath the membrane, and OC = over the external cortex [19]. Each group was evaluated carefully to assess the extension and thickness of the maxillary sinus mucosa. Mucosa thickening was classified as normal < 2 mm, mild 2–4 mm, moderate 4–10 mm, or severe ≥ 10 mm [20]. The correlation between the pathological findings, location of the PSAA, and sinus mucosal thickening was assessed carefully. The ability to detect the location of the PSAA utilizing the AI tool in the eVol DXS software was recorded by each rater and reported as a percentage.

### 2.3. Statistical Analysis

The radiographic data were gathered, organized into tables, and subjected to statistical analysis. Data analysis was performed using the SPSS software, version 25 (IBM Corp. Released 2019. IBM SPSS Statistics for Windows, Version 25.0. Armonk, NY, USA: IBM Corp). The significance of the obtained results was judged at the ≤0.05 level. Crosstabs were utilized to generate the kappa agreement and validity indices. A kappa agreement value of 0.7 was deemed indicative of a strong agreement in the data. The qualitative data were described using frequencies and percentages. The Chi-Square test and Monte Carlo test were used to compare qualitative data between groups as appropriate.

## 3. Results

The sample comprised 120 patients in the study group and 120 patients in the control group. The test group had inflammatory odontogenic lesions in the maxillary premolar and molar region, while the control group was free from any pathological conditions in the maxillary premolar and molar region. Both groups had a safety proximity between the roots’ apices and the sinus floor by safety margins (>2 mm). Each operator achieved separately an excellent 100% detection ability of the PSAA location with the help of the enhanced image features using the AI tool.

**Figure 1:**
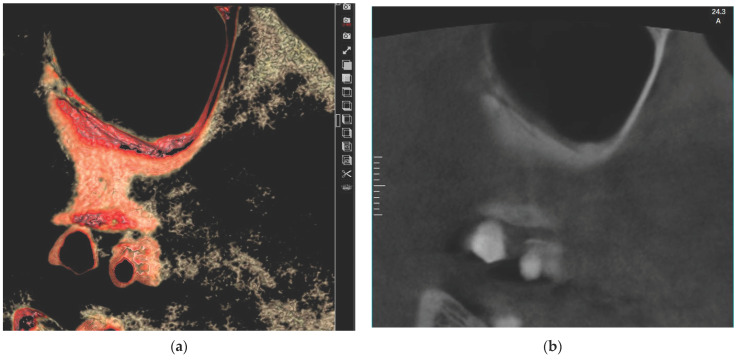
Showing PSAA in intraosseous (IO) location using eVol DXS software where: (**a**) sliced in 3D tool using Sinus filter; (**b**) reconstructed enhanced sagittal cut.

**Figure 2:**
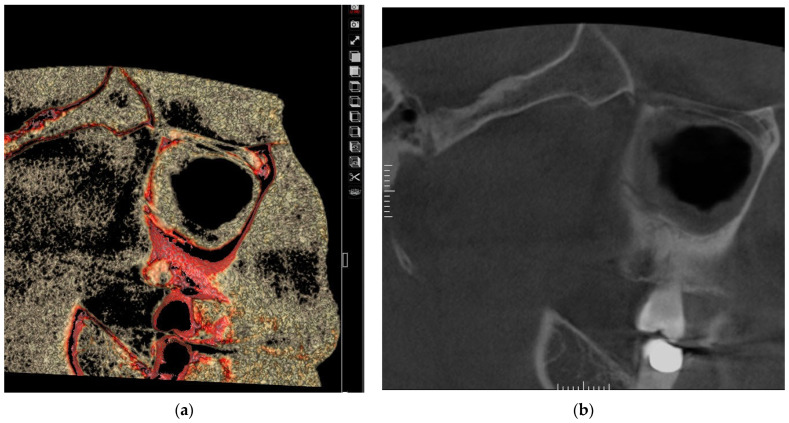
Showing PSAA in beneath the membrane (BM) location using eVol DXS software where: (**a**) sliced in 3D tool using Sinus filter; (**b**) reconstructed enhanced sagittal cut.

**Figure 3:**
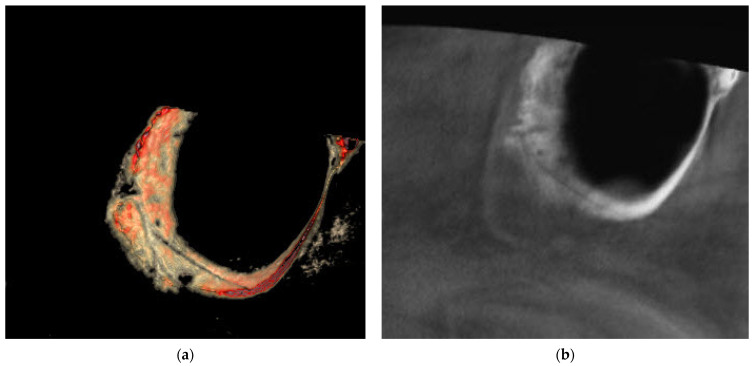
Showing PSAA in over the external cortex (OC) location using eVol DXS software where: (**a**) sliced in 3D tool using Sinus filter; (**b**) reconstructed enhanced sagittal cut.

The results for the association between the maxillary sinus mucosa thickness, which is classified into four categories (normal < 2 mm; mild 2–4 mm; moderate 4–10 mm; and severe ≥ 10 mm), and the three PSAA locations [IO = intraosseous (Figure 1); BM = beneath the membrane (Figure 2); and OC = over the external cortex (Figure 3)] for the test group and control group are presented below. 

**Figure 4:**
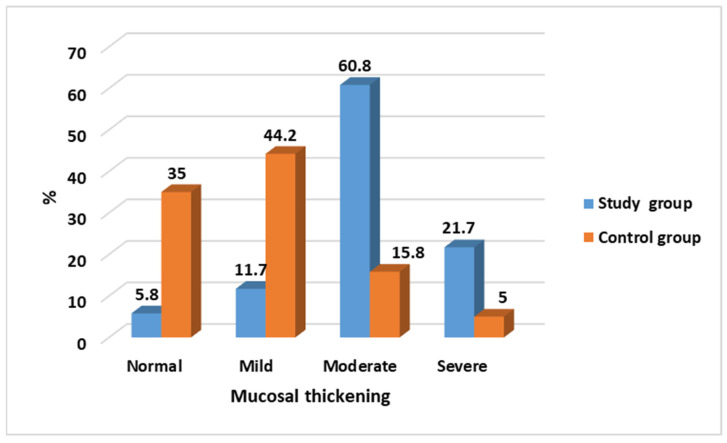
Comparison of mucosal thickening between study and control groups.

The mucosal thickening differs significantly (*p* < 0.001) between the study group and the control group. While in most of the cases it is classified as moderate and severe (60%; 21.7%, respectively), in the control group, an opposite observation is evident with the majority of the cases being normal to mild (35%; 44.2%, respectively), as seen in Table 1 and Figure 4.

**Figure 5:**
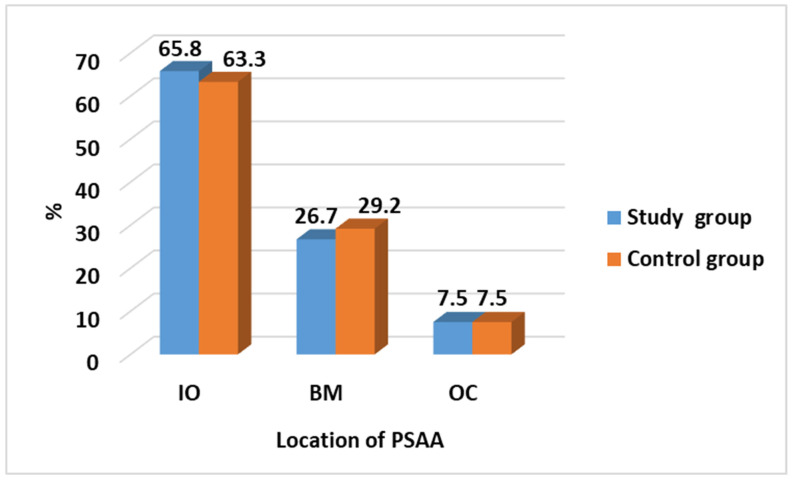
Comparison of location of PSAA between cases and control group.

The Chi-Square test reveals no significant difference between the control group and study group when comparing the location of the PSAA. The observation for the PSAA location shows that 76% of cases or above are located in the IO while only 7.5% of cases are in the OC location, as seen in Table 2 and Figure 5.

There is a statistically significant relation between the location of the PSAA and mucosal thickening among the study group (*p* < 0.001). This relation is also observed and significant in the control group (*p* = 0.009). In the study group, the highest number of cases (*n* = 63) is observed when the PSAA is located in IO with moderate mucosal thickening followed by the BM location with severe mucosal thickening (*n* = 20). However, in the control group, the highest number of cases is reported when the PSAA is located in the IO location, with 37 cases with mild mucosal thickening and 30 cases with normal mucosal thickening, as seen in Table 3 and Table 4.

**Figure 6:**
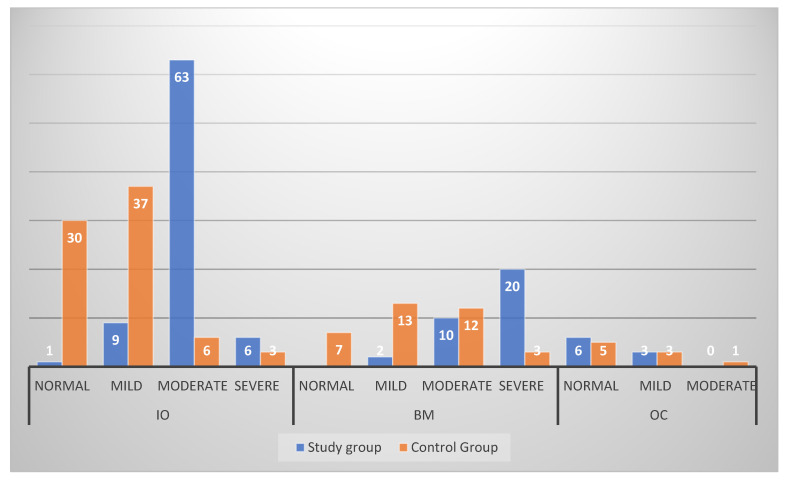
Relation between location of PSAA and mucosal thickening between cases and control group.

When comparing the relation between the location of the PSAA and mucosal thickening between the study group and the control group, it shows that there is a statistically significant difference when the PSAA is located in the IO (*p* < 0.001) or BM (*p* < 0.001) location. This does not apply when the PSAA is located in the OC location, as seen in Table 5 and Figure 6.

## 4. Discussion

The precise determination of the anatomical position of the PSAA holds major importance in dental interventions, particularly those pertaining to the posterior maxillary area. Moreover, it exerts a substantial influence on the health and anatomical structure of the sinus. Multiple studies have demonstrated a positive correlation between the existence of periapical lesions and a higher incidence of mucosal thickening in the maxillary sinus [20,21,22,23,24,25,26]. For instance, an investigation revealed a notable significance in the occurrence of mucosal thickening between individuals with periapical lesions and a control group. The study authors proposed that the presence of periapical lesions may induce inflammation and other alterations, which, in turn, could promote angiogenesis and the development of various structures within the maxillary sinus, ultimately resulting in mucosal membrane thickening [25].

A further investigation revealed a favourable association between the thickness of the mucosal membranes in the maxillary sinus and the magnitude and severity of periapical lesions. This observation implies that the presence of more severe periapical lesions may increase the likelihood of developing mucosal thickening in the maxillary sinus [27]. Based on a comprehensive analysis of the literature in a recent systematic review, it was found that the available evidence substantiates a robust correlation between periapical lesions and the thickness of the mucosal lining in the maxillary sinus. The reviewers emphasized the necessity for more meticulously planned investigations to comprehensively explain the fundamental mechanisms at play and ascertain the optimal strategy for handling this association within the context of therapeutic practice [28]. The results obtained align with the findings of this research. 

In contrast, alternative studies have yielded inconclusive evidence on the correlation between the periapical condition of the posterior maxillary teeth and the occurrence of Schneiderian membrane thickening. The researchers concluded that there is no discernible impact of the proximity of periapical lesions on the presence and the type of inflammatory abnormalities [29,30,31]. Other authors have demonstrated intermediate findings to this end [32,33,34]. So, we concluded that considerable discrepancies in findings could arise when examining the correlation between the thickness of the Schneiderian membrane and the presence, dimensions, and proximity of the periapical lesions due to a multitude of factors, including methodological inconsistencies and variations specific to individual patients. These factors can result from the diversity in imaging techniques (e.g., CBCT, periapical radiography, panoramic imaging), variability in the diagnostic tools, sample characteristics (differences in the characteristics of the study populations, such as age, sex, oral health status, and the prevalence of systemic diseases), or anatomical variations between the study population cases. This means that there are other factors which direct the influence of the inflammatory odontogenic lesions on the maxillary sinus mucosal lining thickening. One of these factors is the location of the PSAA in relation to the walls of the maxillary sinus.

Many studies have focused on the prevalence of the location of the PSAA in relation to the sinus floor [19,35,36,37]. Three main locations can be detected: intraosseous, beneath the membrane, and over the external cortex. In our study, the increased prevalence of sinus thickening in cases where the PSAA is located beneath the sinus membrane emphasizes the need for cautious consideration during dental procedures. Surgical interventions in such cases carry a higher risk of sinus perforation, which can lead to sinusitis or other complications. The prevalence of detecting the location of the PSAA showed great variation in a range between 60 and 92% in most research articles which discussed location issues [7,15,19,37,38]. The ability of AI tools to accurately identify the PSAA’s location preoperatively can greatly assist clinicians in minimizing the potential risks associated with these procedures. In our study, the AI tool in the eVol DXS software enhanced the images, enabling the detection of the location of PSAA in all cases, providing a 100% detection rate. This rate is in accordance with a study by Godil et al. in 2021, which reported a figure of 99.4% [35].

Conversely, the lowest prevalence of sinus thickening was observed when the PSAA was located over the external cortex. This finding suggests a less direct association between the PSAA’s position and the development of sinus pathology. It is possible that the PSAA’s location over the external cortex provides a greater anatomical separation from the sinus mucosa, reducing the likelihood of sinus wall thickening even in the presence of periapical lesions.

While this study demonstrates promising results regarding the utilization of AI tools in detecting PSAA anatomical variations and their impact on sinus wall thickening, there are some limitations to consider. The retrospective nature of the study and the reliance on CBCT images from a single institution may introduce selection bias and limit the generalizability of the findings. The cross-sectional nature of the study is considered another limitation, as longitudinal studies would be more suitable for exploring the temporal relationships between the PSAA’s anatomical location, periapical lesions, and mucosal thickening over time. Further prospective studies involving larger and more diverse patient populations are warranted to validate these results.

## 5. Conclusions

The location of the PSAA has a significant influence on the severity of the mucosal thickening of the maxillary sinus where periapical lesions are present. The highest prevalence was seen when the PSAA was located beneath the sinus membrane. The use of new AI tools offers great potential in dental practice to more effectively detect PSAA anatomical variations and establish a correlation with sinus wall thickening in odontogenic cases. The accurate identification of the PSAA’s location using an AI tool, such as eVol DXS, can enhance image visualization, minimize the risk of complications, and contribute to improved patient outcomes. Further advancements in AI technology and its integration into routine dental care are likely to revolutionize diagnostic and therapeutic approaches in the field of dentistry.

## Figures and Tables

**Table 1:** Comparison of mucosal thickening between study and control groups.

Mucosal Thickening	Study Group *N* = 120 (%)	Control Group *N* = 120 (%)	Test of Significance	Significance for Every Category
Normal	7 (5.8)	42 (35.0)	χ^2^ = 91.89 *p* < 0.001 *	*p* < 0.001 *
Mild	14 (11.7)	53 (44.2)		*p* < 0.001 *
Moderate	73 (60.8)	19 (15.8)		*p* < 0.001 *
Severe	26 (21.7)	6 (5.0)		*p* = 0.001 *

χ^2^: Chi-Square test, * statistically significant.

**Table 2:** Comparison of location of PSAA between study and control groups.

Location of PSAA	Study Group *N* = 120 (%)	Control Group *N* = 120 (%)	Test of Significance	Significance for Every Category
IO	79 (65.8)	76 (63.3)	χ^2^ = 0.192 *p* = 0.908	*p* = 0.686
BM	32 (26.7)	35 (29.2)		*p* = 0.669
OC	9 (7.5)	9 (7.5)		*p* = 1.0

χ^2^: Chi-Square test.

**Table 3:** Relation between location of PSAA and mucosal thickening among study group.

For Study Group	Location of PSAA			
Mucosal Thickening	IO	BM	OC	
Normal	1 (1.3)	0	6 (66.7)	MC = 114.74 *p* < 0.001 *
Mild	9 (11.4)	2 (6.2)	3 (33.3)	
Moderate	63 (79.7)	10 (31.2)	0	
Severe	6 (7.6)	20 (62.5)	0	

* statistically significant.

**Table 4:** Relation between location of PSAA and mucosal thickening among control group.

For Control Group	Location of PSAA			
Mucosal Thickening	IO	BM	OC	
Normal	30 (39.5)	7 (20.0)	5 (55.6)	MC = 16.94 *p* = 0.009 *
Mild	37 (48.7)	13 (37.1)	3 (33.3)	
Moderate	6 (7.9)	12 (34.3)	1 (11.1)	
Severe	3 (3.9)	3 (8.6)	0	

* statistically significant.

**Table 5:** Relation between location of PSAA and mucosal thickening between cases and control group.

Location of PSAA	Mucosal Thickening	Study Group	Control Group	Test of Significance
		*N* = 120 (%)	*N* = 120 (%)	
IO		N = 79 (%)	*N* = 67 (%)	MC = 92.24 *p* < 0.001 *
	Normal	1 (1.3)	30 (39.5)	
	Mild	9 (11.4)	37 (48.7)	
	Moderate	63 (79.7)	6 (7.9)	
	Severe	6 (7.6)	3 (3.9)	
BM		*N* = 32 (%)	*N* = 35 (%)	MC = 27.74 *p* < 0.001 *
	Normal	0	7 (20.0)	
	Mild	2 (6.2)	13 (37.1)	
	Moderate	10 (31.2)	12 (34.3)	
	Severe	20 (62.5)	3 (8.6)	
OC		*N* = 9 (%)	*N* = 9 (%)	MC = 1.09 *p* = 0.580
	Normal	6 (66.7)	5 (55.6)	
	Mild	3 (33.3)	3 (33.3)	
	Moderate	0	1 (11.1)	

* statistically significant.

## Data Availability

The datasets used and/or analyzed during the current study are available from the corresponding author on reasonable request.
